# Dimethyl fumarate promotes the degradation of HNF1B and suppresses the progression of clear cell renal cell carcinoma

**DOI:** 10.1038/s41419-025-07412-7

**Published:** 2025-02-06

**Authors:** Yue Dai, Hongchen Li, Shiyin Fan, Kai Wang, Ziyi Cui, Xinyu Zhao, Xue Sun, Mingen Lin, Jiaxi Li, Yi Gao, Ziyin Tian, Hui Yang, Bingbing Zha, Lei Lv, Yanping Xu

**Affiliations:** 1 Fifth People’s Hospital of Shanghai, MOE Key Laboratory of Metabolism and Molecular Medicine, Department of Biochemistry and Molecular Biology, School of Basic Medical Sciences, Fudan University, Shanghai, China; 2 Tongji Hospital, Frontier Science Center for Stem Cell Research, School of Life Sciences and Technology, Tongji University, Shanghai, China; 3 Department of Endocrinology, Fifth People’s Hospital of Shanghai, Fudan University, Shanghai, China; 4 Department of Neurosurgery, Huashan Hospital, Institute for Translational Brain Research, MOE Frontiers Center for Brain Science, Shanghai Medical College, Fudan University, Shanghai, China

**Keywords:** Urological cancer, Drug discovery, Ubiquitylation, Cancer metabolism

## Abstract

Clear cell renal cell carcinoma (ccRCC) is the most lethal subtype of renal cancer, and its treatment options remain limited. Therefore, there is an urgent need to discover therapeutic agents for ccRCC treatment. Here, we demonstrate that dimethyl fumarate (DMF), an approved medication for multiple sclerosis [1] and psoriasis, can inhibit the proliferation of ccRCC cells. Mechanistically, hepatocyte nuclear factor 1β (HNF1B), a transcription factor highly expressed in ccRCC, is succinated by DMF at cysteine residues, leading to its proteasomal degradation. Furthermore, HNF1B interacts with and stabilizes Yes-associated protein (YAP), thus DMF-mediated HNF1B degradation decreases YAP protein level and the expression of its target genes, resulting in the suppression of ccRCC cell proliferation. Importantly, oral administration of DMF sensitizes ccRCC to sunitinib treatment and enhances its efficacy in mice. In summary, we provide evidences supporting DMF as a potential drug for clinical treatment of ccRCC by targeting HNF1B and reveal a previously unrecognized role of HNF1B in regulating YAP in ccRCC.

## Introduction

Renal cell carcinoma (RCC) which arises from renal tubular epithelial cells, is one of the malignant tumors of the urinary system. According to its pathological features, RCC primarily consists of three subtypes: chromophobe renal cell carcinoma (chRCC), clear cell renal cell carcinoma (ccRCC) and papillary renal cell carcinoma (pRCC). Among these, ccRCC is the most prevalent subtype and is characterized by the presence of abundant cytoplasmic glycogen and lipids [2, 3]. ccRCC accounts for ~75–80% of all RCC cases, and has a poorer prognosis compared to other subtypes [4]. Over the past few decades, various therapeutic strategies have been employed for ccRCC treatment, including surgical management, chemotherapy and radiotherapy [5]. Among these treatments, surgery remains the primary treatment option for ccRCC due to its limited response to chemotherapy and radiotherapy. Since early-stage ccRCC often presents with few noticeable clinical symptoms, and in several cases, metastasis is already present at the time of diagnosis, which contributes to a poor prognosis with the 5-year survival rate is only 10% [6–8]. Therefore, it is crucial to develop new strategies for ccRCC treatment.

HNF1B, a 65-kDa protein, is a member of the superfamily of transcription factors that includes homeodomain proteins such as Pit-1, Oct-1/2, and POU. HNF1B consists of three functional regions: an amino-terminal domain, a DNA-binding domain and a carboxy-terminal domain. The gene encoding HNF1B is located on the long arm of chromosome 17 [9–11]. During the early embryonic development, the expression of HNF1B is expressed in several organs, including liver, kidney, pancreas, lung and urinary tract. Heterozygous mutations in *HNF1B* are associated with several congenital diseases including renal cysts, pancreatic hypoplasia, abnormal liver function tests, and urogenital tract abnormalities, suggesting that HNF1B is crucial for the normal development of these organs [1, 12]. Although research on HNF1B has increased in recent years, its precise role in carcinogenesis remains insufficiently understood. Interestingly, HNF1B functions as a protooncogene in some tumors, while it acts as a tumor suppressor in others, and whether it behaves as an oncogene or tumor suppressor appears to depend on the type and histogenesis of the tumor [13, 14].

Dimethyl fumarate (DMF) is a small molecule compound, a dimethyl ester of fumaric acid, which was first approved by the U.S. Food and Drug Administration in 2013 under the brand name Tecfidera [15]. It is registered as an anti-inflammatory drug for the treatment of autoimmune diseases, such as multiple sclerosis and psoriasis [16, 17]. Several studies have demonstrated that the molecular mechanisms underlying these effects can be mainly attributed to two aspects. First, DMF disrupts the interaction between Kelch-like ECH-associated protein 1 (KEAP1) and erythroid 2–related factor 2 (Nrf2), leading to activation of Nrf2 and subsequent expression of antioxidant genes, which protects cells from reactive oxygen species that is generated during inflammation [16, 18, 19]. Second, DMF inhibits NF-κB signaling pathway by suppressing the translocation of NF-κB family members into the nucleus, resulting in a significant decrease in the production of proinflammatory cytokines [16, 17, 20, 21]. However, the function of DMF in ccRCC and its underlying mechanisms remain unknown. The present study uncovers a previously unknown role of DMF in ccRCC, embodying the concept of “old drugs, new uses”, which offers a more time- and cost-efficient alternative compared with the new drug discovery [22, 23]. We demonstrate that DMF targets HNF1B for ubiquitin-dependent proteasomal degradation and effectively suppresses the proliferation of ccRCC cells by impairing HNF1B mediated stabilization of Yes-associated protein (YAP). Notably, oral administration of DMF decreases HNF1B levels in mice and enhances the sensitivity of ccRCC to sunitinib treatment. Overall, our study unveils a new regulatory axis involving DMF, HNF1B and YAP, and demonstrates that DMF is a potential candidate for clinical treatment of ccRCC.

## Results

### HNF1B facilitates cell proliferation in ccRCC

**Fig. 1:**
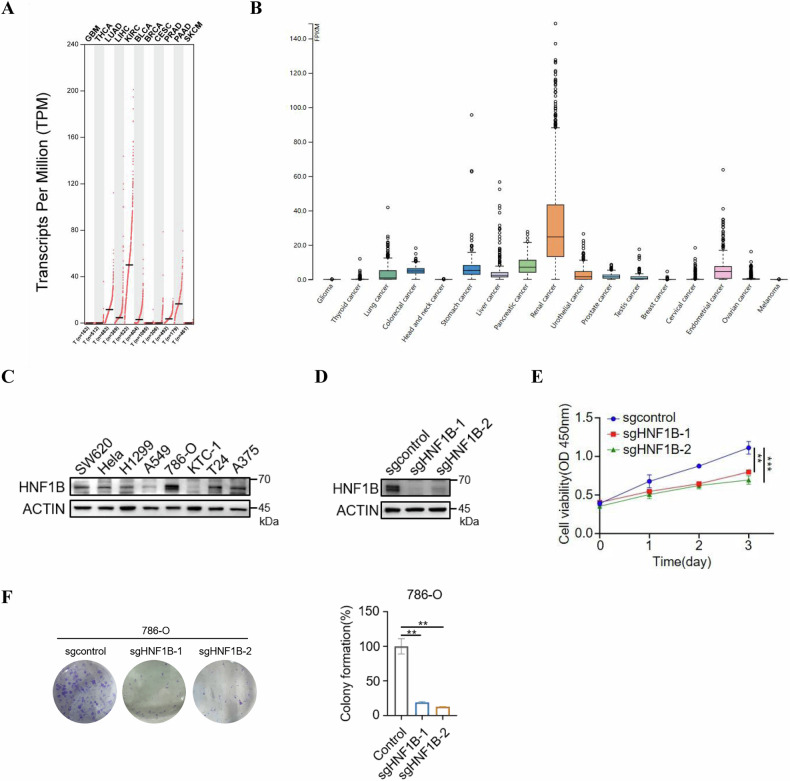
HFN1B positively regulates cell proliferation in ccRCC. **A** HNF1B expression analysis of different cancers using GEPIA database. **B** HNF1B expression analysis of different cancers using The Human Protein Atlas database. **C** Western blot analysis of HNF1B in various cancer cell lines. **D** CRISPR/Cas9 system was employed to knock out HNF1B in 786-O cells and HNF1B KO efficiency was verified by Western blot analysis. **E** The proliferation of 786-O cells with HNF1B knockout was determined using CCK-8 assays. The data represent the mean ± SD of *n* = 3 independent experiments. Statistical differences were determined by one-way ANOVA. **P < 0.01, ***P < 0.001. **F** Colony formation assays and statistical analysis of the proliferation of 786-O cells with HNF1B knockout. The data represent the mean ± SD of *n* = 3 independent experiments. Statistical differences were determined by one-way ANOVA. **P < 0.01.

To investigate the role of HNF1B in tumorigenesis, we utilized the GEPIA and HPA databases to determine the expression level of HNF1B in various types of tumors. Notably, HNF1B shows the highest expression in ccRCC compared to other cancers across both databases (Fig. 1A, B). Consistently, the protein level of HNF1B is also highest in 786-O ccRCC cell line compared to other cancer cell lines, such as SW620 (colorectal cancer), HeLa (cervical carcinoma), KTC-1 (thyroid cancer), T24 (bladder cancer), A375 (melanoma), H1299 and A549 (non-small cell lung cancer) (Fig. 1C). A previous study reported that HNF1B functions as a tumor suppressor and inhibits cell proliferation in prostate cancer [24]. However, another study suggested that HNF1B may act as an oncogene, even though the underlying mechanism remains unclear [25]. Given these findings, we wanted to explore what role did HNF1B play in the cell proliferation of ccRCC. HNF1B knockout cells were generated using the CRISPR/Cas9 system (Fig. 1D), and we found that HNF1B knockout significantly suppressed both proliferation and colony formation of ccRCC cells (Fig. 1E, F). Together, these results indicate that HNF1B positively regulates cell proliferation in ccRCC.

### DMF suppresses cell proliferation and decreases HNF1B protein level in ccRCC

**Fig. 2:**
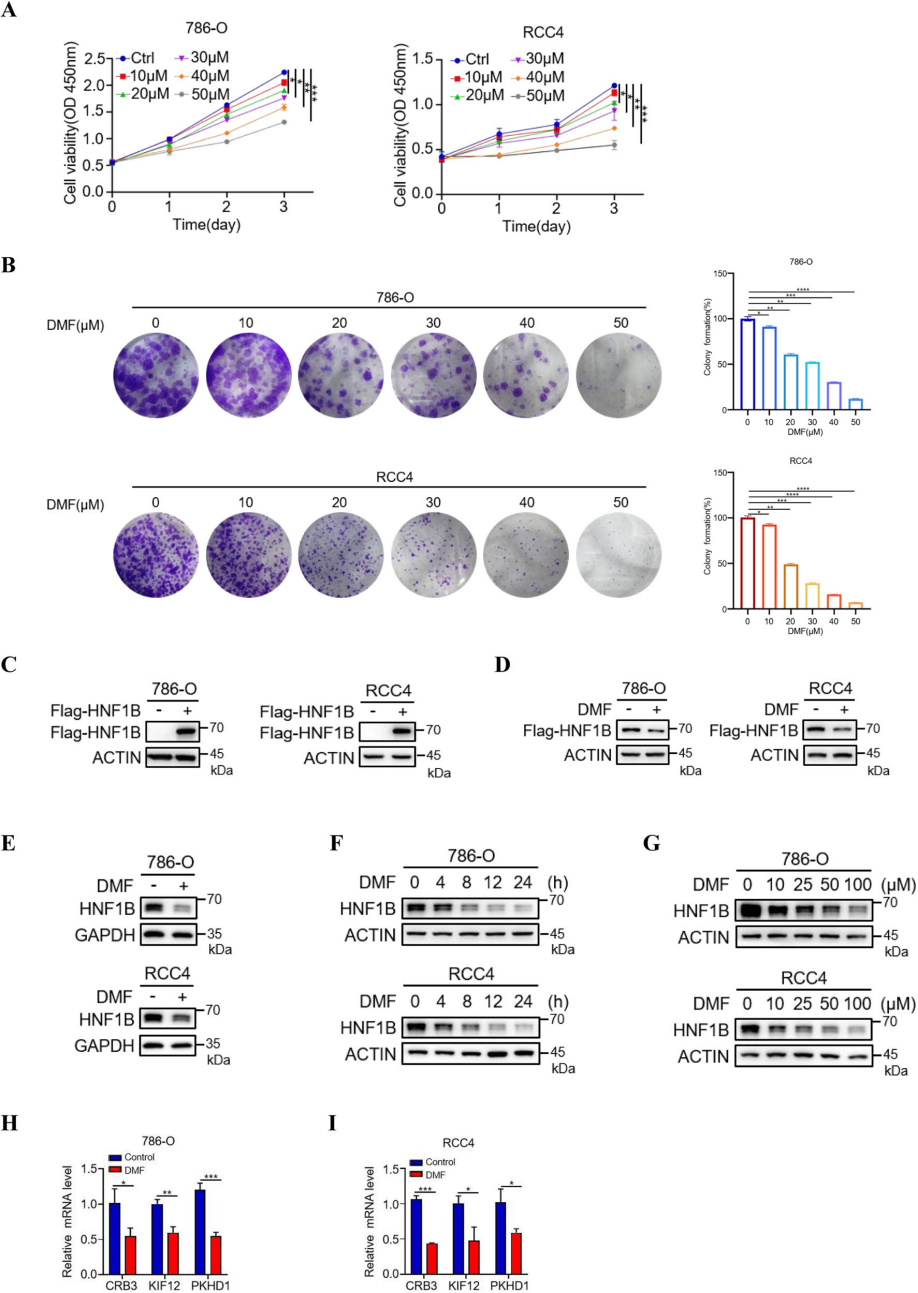
DMF suppresses cell proliferation and decreases HNF1B protein level. **A** CCK-8 assays of the proliferation of 786-O and RCC4 cells treated with a serial dose of DMF as indicated. The data represent the mean ± SD of *n* = 3 independent experiments. Statistical differences were determined by one-way ANOVA. *P < 0.05, **P < 0.01, ***P < 0.001. **B** Colony formation assays and statistical analysis of the proliferation of 786-O and RCC4 cells treated with a serial dose of DMF as indicated. The data represent the mean ± SD of *n* = 3 independent experiments. Statistical differences were determined by one-way ANOVA. *P < 0.05, **P < 0.01, ***P < 0.001, ***P < 0.0001. **C** Western blot analysis of 786-O and RCC4 cells stably overexpressing Flag-HNF1B. **D** Western blot analysis of exogenous Flag-HNF1B in 786-O and RCC4 cells treated with or without DMF (50 μM, 12 h). **E** Western blot analysis of endogenous HNF1B in 786-O and RCC4 cells treated with or without DMF (50 μM, 12 h). **F** Western blot analysis of HNF1B treated with 50 μM DMF for different times as indicated. **G** Western blot analysis of HNF1B treated with increasing concentrations of DMF (0 to 100 μM) for 12 h. qPCR analysis of *CRB3*, *KIF12* and *PKHD1* levels in 786-O (**H**) and RCC4 (**I**) cells treated with or without 50 μM DMF for 12 h.

Previous studies reported that DMF inhibits breast cancer and melanoma, which prompted us to investigate if DMF has the same impact on ccRCC. Indeed, we observed that DMF significantly inhibited the proliferation and colony formation of 786-O and RCC4 cells in a dose-dependent manner (Fig. 2A, B). Given the high expression of HNF1B in ccRCC and HNF1B knock out negatively regulates cell proliferation of ccRCC, we next explored whether the impact of DMF on cell proliferation was related to HNF1B. We generated ccRCC cells stably expressing Flag-HNF1B (Fig. 2C), treated these cells with DMF, and examined the effect of DMF on exogenous Flag-HNF1B. The results showed that exogenous Flag-HNF1B protein level was significantly reduced upon DMF treatment (Fig. 2D). Furthermore, we assessed the expression of endogenous HNF1B after DMF treatment and it showed that DMF also decreased endogenous HNF1B protein level in both 786-O and RCC4 cells (Fig. 2E). To further confirm these results, we treated these two cell lines with DMF for varying durations and found that DMF reduced HNF1B levels in a time-dependent manner (Fig. 2F). Similar effects were observed when cells were treated with increasing concentrations of DMF, and the expression of HNF1B decreased in a dose-dependent manner (Fig. 2G). To further validate the effects of DMF on HNF1B, we examined the expression levels of HNF1B target genes under DMF treatment. The results revealed that DMF significantly downregulated the mRNA levels of *CRB3*, *KIF12* and *PKHD1* in 786-O and RCC4 cells (Fig. 2H, I), further confirming the role of DMF on the downregulation of HNF1B. Together, these data indicate that DMF treatment decreases HNF1B protein levels in ccRCC.

### DMF downregulates HNF1B via ubiquitin-proteasome pathway

**Fig. 3:**
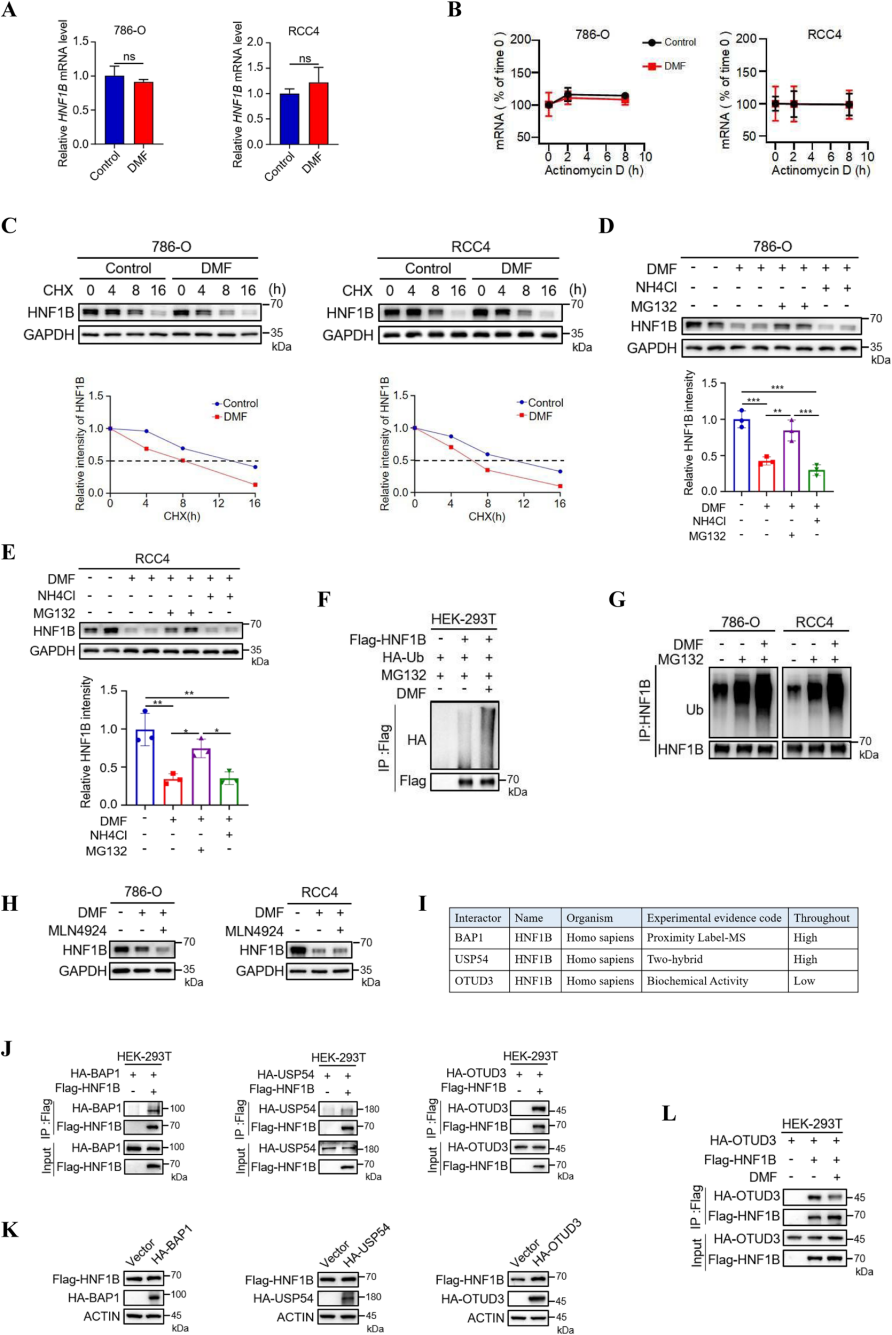
Ubiquitin-proteasome pathway contributes to DMF-induced downregulation of HNF1B. **A** qPCR analysis of *HNF1B* level in 786-O and RCC4 cells treated with or without 50 μM DMF for 12 h. **B** *HNF1B* mRNA stability under DMF treatment (50 μM) was determined in 786-O and RCC4 cells treated with 5 μg/mL actinomycin D for different time points as indicated. *n* = 3 biologically independent experiments per group. **C** The half-life of HNF1B protein under DMF treatment was determined in the presence of 100 μg/mL cycloheximide (CHX) in 786-O and RCC4 cells. Western blot analysis of HNF1B level in 786-O (**D**) and RCC4 (**E**) cells treated with DMF in the absence or presence of lysosome inhibitor NH_4_Cl (20 mM, 12 h), or proteasome inhibitor MG132 (10 μM, 12 h). The quantifications are shown below. The data represent the mean ± SD of *n* = 3 independent experiments. Statistical differences were determined by one-way ANOVA. *P < 0.05, **P < 0.01, ***P < 0.001. **F** Flag-HNF1B and HA-Ub were co-transfected into HEK-293T cells with or without DMF treatment. HNF1B ubiquitination level was determined by western blot analysis. **G** Western blot analysis of endogenous HNF1B ubiquitination level in 786-O and RCC4 cells treated with DMF treatment. **H** Western blot analysis of HNF1B level in 786-O and RCC4 cells treated with DMF in the absence or presence of 0.5 μM MLN4924. **I** Identification of potential HNF1B-interacting deubiquitinases using BioGRID. **J** HEK-293T cells were transfected with indicated constructs for 48 h. Cells were collected for coimmunoprecipitation analysis. **K** Western blot analysis of Flag-HNF1B in HEK-293T cells stably expressing Flag-hnf1b transfected with HA-BAP1, HA-USP54, or HA-OTUD3. **L** Co-IP analysis of interactions between Flag-HNF1B and HA-OTUD3 in HEK-293T cells with or without DMF treatment.

To determine how DMF decreases the expression level of HNF1B, we examined the effects of DMF treatment on the mRNA level of *HNF1B* in ccRCC cells, and there was no alteration in *HNF1B* mRNA level upon DMF treatment (Fig. 3A). Furthermore, DMF has no effect on the mRNA stability of HNF1B (Fig. 3B), indicating that DMF did not regulate HNF1B expression through transcription. Interestingly, in the presence of cycloheximide, a protein synthesis inhibitor, the half-life of HNF1B under DMF treatment was shorter than that of the control group in both 786-O and RCC4 cells (Fig. 3C), suggesting that DMF may reduce the protein stability of HNF1B. To investigate the mechanism how HNF1B was degraded, we treated cells with NH4Cl, a lysosome pathway inhibitor and MG132, a proteasome pathway inhibitor, respectively, to determine which inhibitor could block HNF1B degradation in the presence of DMF. The results showed that only MG132 could rescue DMF-induced degradation of HNF1B, suggesting that DMF reduces HNF1B protein level via the ubiquitin-proteasome pathway (Fig. 3D, E). To confirm this hypothesis, we examined the ubiquitination level of HNF1B in HEK-293T, 786-O and RCC4 cells, and found that both exogenous and endogenous HNF1B ubiquitination levels significantly increased upon DMF treatment (Fig. 3F, G). We next determined whether MLN4924, an inhibitor of cullin-RING E3 ligases, plays a role in DMF-induced HNF1B degradation and found that it did not rescue the expression levels of HNF1B in the presence of DMF (Fig. 3H). We then explored the potential deubiquitinases (DUBs) that may contribute to the DMF induced reduction of HNF1B. Analysis of the BioGRID database revealed three DUBs—BAP1, USP54 and OTUD3—that show potential interactions with HNF1B (Fig. 3I). We then determined the interactions of Flag-HNF1B with these three DUBs via coimmunoprecipitation (co-IP) in HEK-293T cells. The results confirmed the interactions between Flag-HNF1B and these three DUBs with different degrees (Fig. 3J). Moreover, overexpression of HA-OTUD3 increased the protein levels of Flag-HNF1B, while HA-BAP1 and HA-USP54 had no obvious effects (Fig. 3K), indicating that HA-OTUD3 may play a crucial role in HNF1B stability regulation upon DMF treatment. To confirm this hypothesis, we performed further co-IP assays and found that DMF treatment downregulated the interaction between Flag-HNF1B and HA-OTUD3 (Fig. 3L). Collectively, these data suggest that OTUD3 binds to and stabilizes HNF1B, and that DMF treatment impairs this interaction, thereby promoting the degradation of HNF1B via ubiquitin-proteasome pathway.

### DMF-induced HNF1B succination enhances its ubiquitination

**Fig. 4:**
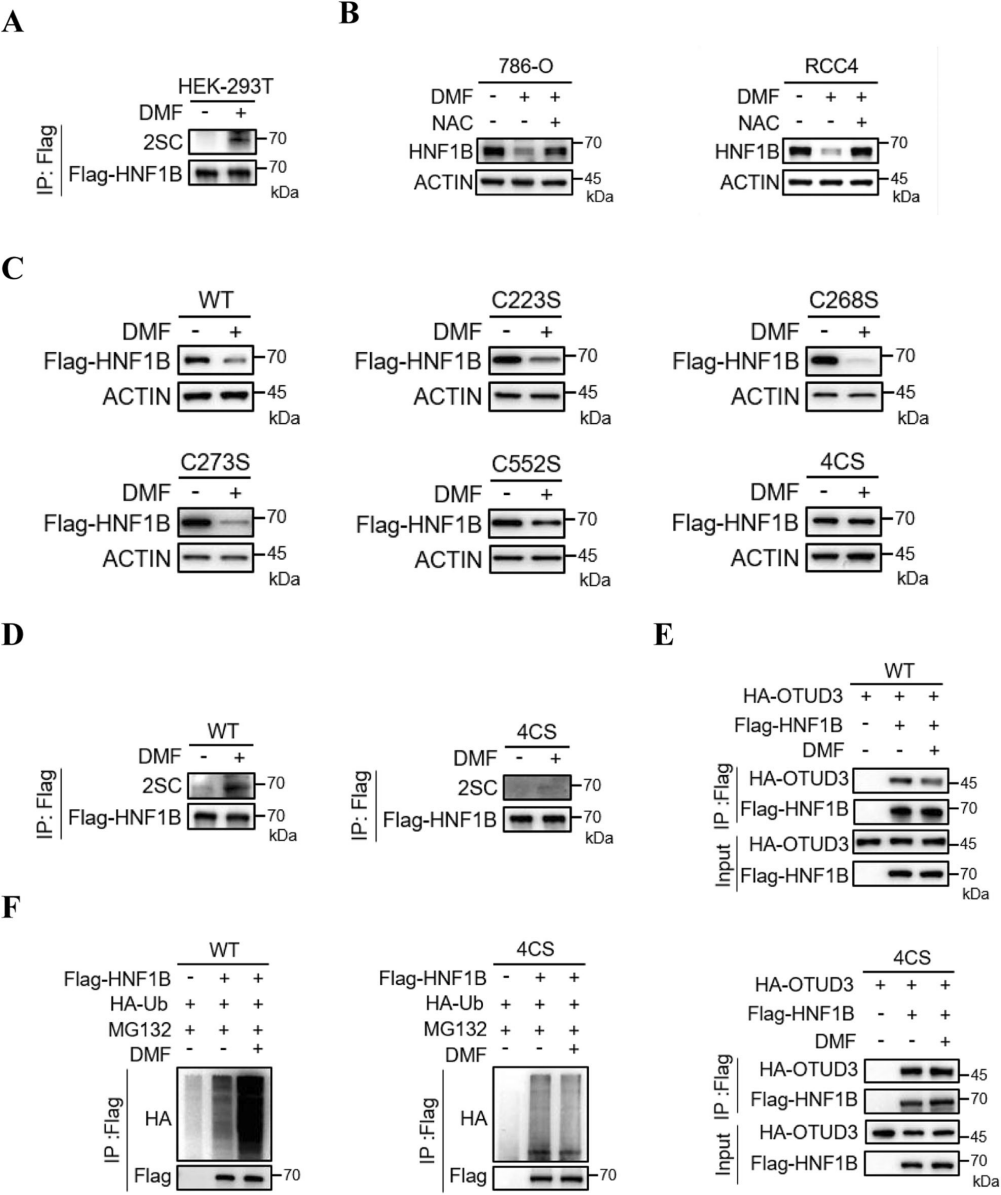
HNF1B succination increases its ubiquitination. **A** Co-IP analysis of Flag-HNF1B succination in HEK-293T cells transfected with Flag-HNF1B with or without DMF treatment. **B** The effect of N-acetyl-L-cysteine (NAC) on DMF-induced HNF1B degradation was determined by western blot. Cells were pretreated with 5 mM NAC for 1 h before treated with DMF (50 μM, 12 h). **C** Western blot analysis of Flag-HNF1B derived from 786-O cells stably expressing WT, C223S, C268S, C273S, C552S or 4CS of HNF1B treated with or without DMF (50 μM, 12 h). **D** Succination of WT or 4CS mutant of HNF1B were analyzed by co-IP assay in HEK-293T cells transfected with indicated plasmids. **E** The effects of DMF on the interaction between HNF1B WT or 4CS mutant and HA-OTUD3 were determined by co-IP and western blot. **F** HEK-293T cells were transfected with HA-Ub and Flag-HNF1B WT or 4CS mutant in the presence or absence of DMF. Ubiquitination levels of WT HNF1B and 4CS mutant were analyzed by co-IP and western blot.

As a thiol-reactive electrophile, DMF can induce covalent modifications of thiols and directly reacts with cysteine residues of different kinds of proteins, including KEAP1, NF-κB, LYN and GSDMD, leading to the inactivation of these proteins [21, 26–28]. We hypothesized that DMF may also affect HNF1B by modification of its cysteine residues. Indeed, HNF1B was identified as a target of succination following DMF treatment (Fig. 4A). To investigate this further, we pretreated cells with N-acetyl-L-cysteine (NAC), a cell-permeable thiol that mimics the role of cysteines, prior to DMF treatment [29, 30]. Interestingly, NAC suppressed DMF induced decrease of HNF1B protein levels in both 786-O and RCC4 cells, suggesting that NAC may protect the cysteines of HNF1B from modification by DMF, thereby preventing subsequent ubiquitination and degradation of HNF1B (Fig. 4B). To determine which cysteine residues of HNF1B are succinated by DMF and contribute to its degradation, we generated cysteine mutants of HNF1B and assessed the effects of DMF on these mutants. Surprisingly, the protein levels of wild-type (WT) and C223S, C268S, C273S and C552S HNF1B mutants all decreased under DMF treatment, while the 4 CS mutant exhibited no response to DMF (Fig. 4C). More importantly, unlike the WT form, the 4CS mutant HNF1B could not be succinated by DMF (Fig. 4D), and DMF impaired the interaction between OTUD3 and WT HNF1B, while there was no detectable change in the interaction between OTUD3 and 4CS mutant HNF1B upon DMF treatment (Fig. 4E). Consistently, DMF significantly increased the ubiquitination level of WT HNF1B, while the ubiquitination level of 4CS mutant HNF1B did not change under DMF treatment (Fig. 4F). Collectively, these results indicate that DMF promotes succination of HNF1B, impairing its interaction with OTUD3, which in turn enhances its ubiquitination and degradation.

### DMF-HNF1B axis suppresses YAP activity

**Fig. 5:**
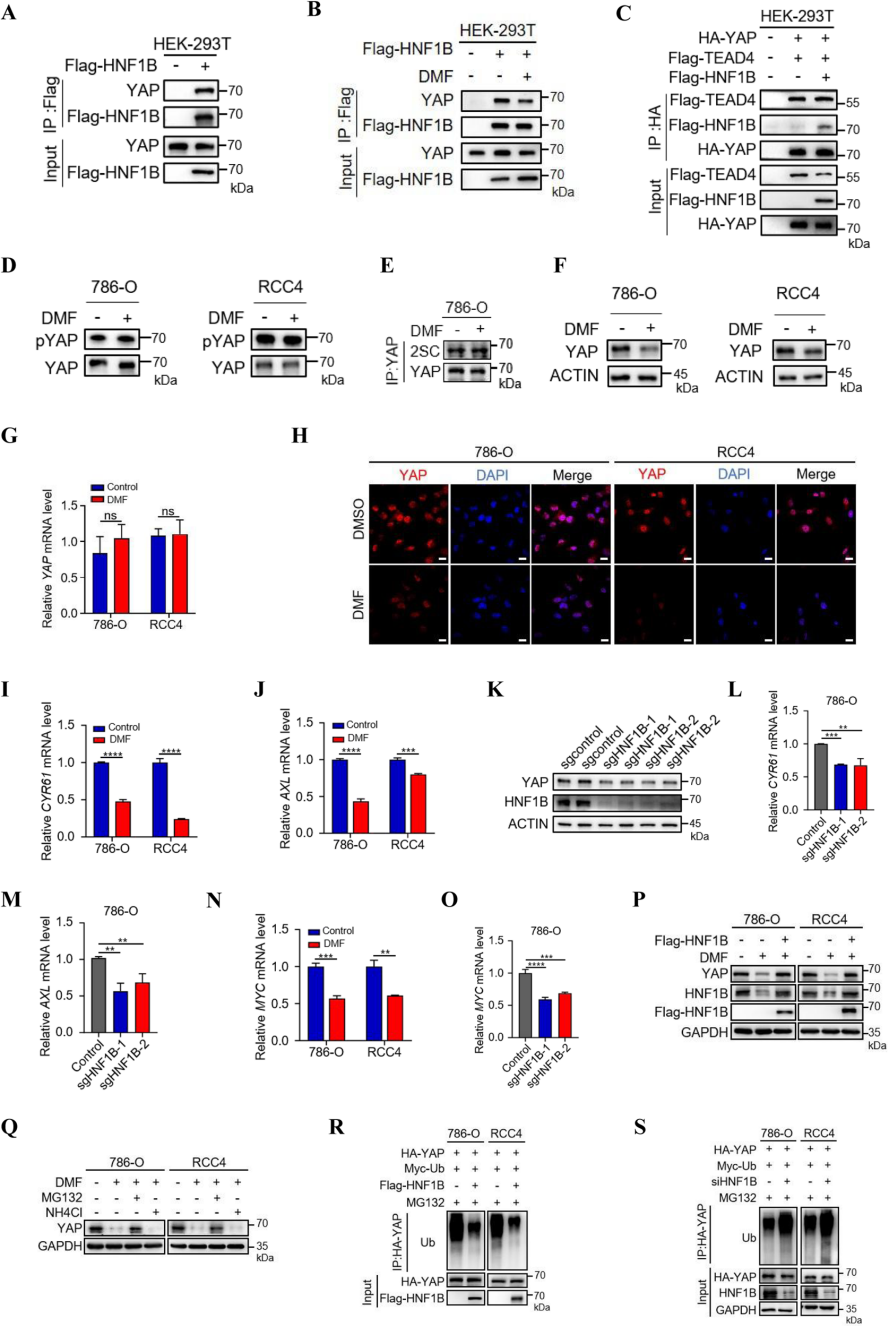
DMF-HNF1B axis suppresses YAP activity. **A** Co-IP of exogenous Flag-HNF1B and endogenous YAP in HEK-293T cells. **B** The effect of DMF on the interaction of Flag-HNF1B and YAP was performed by co-IP and western blot. **C** The effect of overexpressed HNF1B on the interaction between YAP and TEAD4. HEK-293T cells with or without overexpression of Flag-HNF1B were transfected with HA-YAP and Flag-TEAD4 and subjected to Co-IP. **D** Western blot analysis of pYAP in ccRCC cells with or without DMF treatment. **E** Succination of endougenous YAP was analyzed by co-IP followed by western blot in 786-O cells with or without DMF treatment. **F** Western blot analysis of YAP in ccRCC cells with or without DMF treatment. **G** qPCR analysis of *YAP* mRNA levels in 786-O and RCC4 cells treated with DMF. The data represent the mean ± SD of *n* = 3 independent experiments. **H** Immunofluorescence staining assay of YAP expression in 786-O and RCC4 cells. Red, YAP, blue, DAPI. Scale bars, 20 μm. qPCR analysis of relative *CYR61* (**I**) and *AXL* (**J**) mRNA level derived from ccRCC cells treated with or without DMF. The data represent the mean ± SD of *n* = 3 independent experiments. Statistical differences were determined using Student’s *t*-test. ***P < 0.001, ****P < 0.0001. **K** Western blot analysis of YAP in HNF1B KO 786-O cells. qPCR analysis of relative *CYR61* (**L**) and *AXL* (**M**) mRNA level derived from HNF1B KO 786-O cells. The data represent the mean ± SD of *n* = 3 independent experiments. Statistical differences were determined using ordinary one-way ANOVA. **P < 0.01, ***P < 0.001. **N** qPCR analysis of *MYC* mRNA level derived from786-O and RCC4 cells treated with or without DMF. The data represent the mean ± SD of *n* = 3 independent experiments. Statistical differences were determined using Student’s *t*-test. **P < 0.01, ***P < 0.001. **O** qPCR analysis of *MYC* mRNA levels in control and HNF1B KO 786-O cells. The data represent the mean ± SD of *n* = 3 independent experiments. **P** Western blot analysis of YAP levels in 786-O and RCC4 cells treated with DMF and transfected with Flag-HNF1B as indicated. **Q** Western blot analysis of YAP levels in 786-O and RCC4 cells treated with DMF in the absence or presence of proteasome inhibitor MG132 (10 μM, 12 h), or lysosome inhibitor NH_4_Cl (20 mM, 12 h). **R** Western blot analysis of YAP ubiquitination levels in 786-O and RCC4 cells transfected with or without Flag-HNF1B. **S** Western blot analysis of YAP ubiquitination levels in control and HNF1B knockdown 786-O and RCC4 cells. Statistical differences were determined using ordinary one-way ANOVA. ***P < 0.001, ****P < 0.0001.

It is well established that YAP plays a critical role in promoting cell proliferation, metastasis and chemoresistance in multiple solid tumors [31]. Notably, HNF1B was identified as a potential interacting protein of YAP by mass spectrum [32], which prompted us to investigate whether HNF1B inhibits cell proliferation through YAP. To examine this hypothesis, we conducted a co-IP assay, which revealed that HNF1B indeed interacted with YAP (Fig. 5A). Interestingly, this interaction could be inhibited by DMF (Fig. 5B). Given that the cooperation of YAP-TEAD4 complex is enhanced by HHEX, which promoted cell proliferation [33], we examined whether HNF1B plays a similar role, and found that HNF1B did not affect the interaction between YAP and TEAD4 (Fig. 5C). Moreover, we examined the effects of DMF on YAP phosphorylation and succination levels and results showed that there were no significant changes upon DMF treatment (Fig. 5D, E). Surprisingly, we observed that DMF downregulated YAP protein levels, but not its mRNA levels, in both 786-O and RCC4 cells (Fig. 5F, G). Consistently, immunofluorescence assays also revealed that DMF decreased the protein levels of YAP both in cytoplasm and nucleus (Fig. 5H). Moreover, the mRNA levels of *CYR61* and *AXL*, the downstream target genes of YAP, were also decreased following DMF treatment (Fig. 5I, J). We further determined the expression level of YAP in HNF1B knockout cells. Western blot analysis demonstrated that YAP expression level was reduced in the absence of HNF1B (Fig. 5K). Notably, qPCR analysis revealed that HNF1B knockout also significantly decreased YAP target gene expression (Fig. 5L, M). These data suggest that DMF-induced HNF1B degradation may trigger YAP instability, leading to the inactivation of YAP downstream targets. A previous study reported that YAP can act as a transcriptional regulator of MYC, which in turn controls cell cycle [34]. We therefore evaluated the impact of DMF on MYC mRNA levels via qPCR. Our findings revealed a marked reduction in MYC mRNA levels after DMF treatment (Fig. 5N), with a similar outcome observed in HNF1B knockout cells (Fig. 5O). Overall, these findings demonstrate that DMF promotes HNF1B degradation, leading to the suppression of YAP and its downstream target gene expression.

Next, we determine how DMF regulates YAP stability and results showed that DMF mediated YAP degradation can be rescued by the overexpression of HNF1B in ccRCC cells (Fig. 5P), suggesting DMF mediated YAP degradation is dependent on HNF1B. To confirm which degradation pathway was involved in this process, we treated cells with MG132 or NH4Cl in the presence of DMF. The results showed that only MG132 could rescue DMF-induced degradation of YAP, suggesting that DMF reduces YAP protein level via the ubiquitin-proteasome pathway (Fig. 5Q). Furthermore, overexpression of HNF1B significantly decreased YAP ubiquitination levels (Fig. 5R), while knocking down HNF1B led to an increase of its ubiquitination in both 786-O and RCC4 cells (Fig. 5S). Taken together, these results demonstrate that HNF1B decreases the ubiquitination level of YAP and enhances its stability, whereas DMF mediated HNF1B succination promotes YAP ubiquitination and degradation.

### DMF enhances the sensitivity of ccRCC cells to sunitinib

**Fig. 6:**
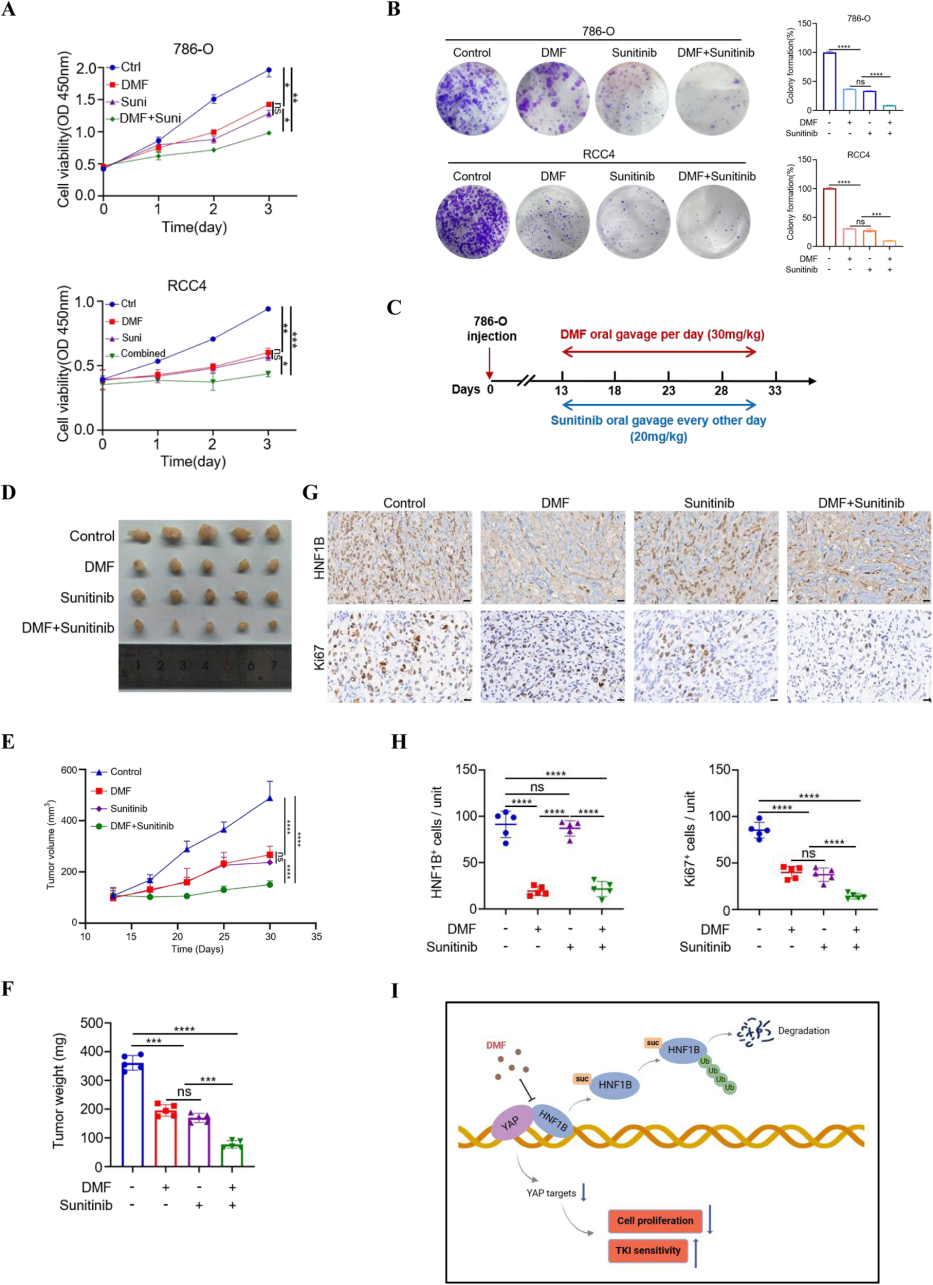
DMF enhances sensitivity of ccRCC to sunitinib treatment. **A** 786-O and RCC4 cells were individually divided into four groups, treated with vehicle, DMF, sunitinib, DMF plus sunitinib, respectively, then subjected to CCK-8 analysis to determine cell proliferation. **B** Colony formation assays and statistical analysis of the proliferation of 786-O and RCC4 cells treated with vehicle, DMF, sunitinib, DMF plus sunitinib, respectively. The data represent the mean ± SD of *n* = 3 independent experiments. Statistical differences were determined using ordinary one-way ANOVA. *P < 0.05, **P < 0.01, ***P < 0.001, ****P < 0.0001, ns, not significant. **C** Schematic representation of the animal experiment. **D** 786-O cells were employed to mouse xenograft model and the mouse was administrated with DMF and/or sunitinib as indicated. Tumors were dissected from each group. **E** Tumor growth of each group was monitored. Statistical differences were determined using ordinary one-way ANOVA. ****P < 0.0001, ns, not significant. **F** The weight of tumors from each group was analyzed. Statistical differences were determined using ordinary one-way ANOVA. ***P < 0.001, ****P < 0.0001, ns, not significant. **G** IHC staining of HNF1B and Ki67 in xenograft tumors. **H** Quantifications of IHC images. Statistical differences were determined using ordinary one-way ANOVA. ****P < 0.0001, ns, not significant. **I** Working model depicting the mechanism of DMF-mediated HNF1B degradation and ccRCC inhibition.

Sunitinib, a tyrosine kinase inhibitor (TKI), has been approved as a first-line treatment for ccRCC. Recently, several studies have associated YAP activation with resistance to sunitinib therapy, presenting a major obstacle to improve survival of patients with ccRCC [35]. Based on our finding that DMF reduced HNF1B mediated YAP stabilization, we wonder whether DMF can enhance the sensitivity of ccRCC to sunitinib treatment. The results showed that treatment with DMF or sunitinib alone moderately inhibited cell proliferation and colony formation. Notably, the combination of DMF and sunitinib exhibited a strong synergistic effect on both cell proliferation and colony formation (Fig. 6A, B).

To further validate these findings in vivo, we employed a cell-derived xenograft tumor model. 786-O cells were injected subcutaneously into mice, which were then treated with DMF and/or sunitinib once the tumors became palpable (Fig. 6C). Consistent with the in vitro observations, the combination of DMF and sunitinib significantly suppressed tumor growth and reduced tumor weight compared to single-agent treatments (Fig. 6D–F). Immunohistochemistry (IHC) staining revealed that oral administration of DMF significantly decreased the protein levels of tumoral HNF1B (Fig. 6G, H). Moreover, the combined treatment with DMF and sunitinib dramatically reduced Ki67 levels in tumor tissues (Fig. 6G, H). Collectively, these data reveal an important role of DMF in enhancing the sensitivity of ccRCC to sunitinib treatment (Fig. 6I).

## Discussion

Despite the availability of several treatment options for ccRCC, patients remain susceptible to develop drug resistance, which is a key factor in disease progression and contributes to high global mortality rates. As a result, there is a critical need to explore novel therapeutic drugs and strategies. However, drug discovery is an expensive and time-consuming process, typically costing around $2 billion and taking over a decade, with most drugs failing in clinical trials due to inadequate efficacy or adverse side effects [36]. Recently, drug repurposing, granting approved drugs new therapeutic uses, has garnered significant attention. Researchers have identified several existing drugs with new applications in other diseases. For example, metformin, a first-line drug for the treatment of type 2 diabetes mellitus, possesses immunomodulatory properties in cancer and inflammatory diseases, while chloroquine, known as an anti-malaria medication, shows anti-tumor activity in several types of cancer [37, 38]. DMF has been approved for the treatment of MS and psoriasis for several years, and in the current study, we identified its novel role in ccRCC therapy. As shown in Fig. 6I, DMF succinates HNF1B, leading to its proteasomal degradation and subsequent inactivation of YAP, which inhibits cell proliferation of ccRCC. This suggests that DMF is a promising candidate for ccRCC treatment, given that its pharmacokinetics and safety profile have been well-established.

ccRCC is characterized by mutations of Von Hippel–Lindau (*VHL*), which lead to abnormal activation of hypoxia inducible factors, a group of transcription factors that regulate various genes, including vascular endothelial growth factor (VEGF), facilitating neovasculogenesis in tumors [2, 39]. Sunitinib, one of the TKIs, was used to target VEGF signaling pathway for the treatment of ccRCC [39]. However, most of ccRCC patients can develop resistance to sunitinib treatment. In this study, we demonstrated that DMF can enhance the sensitivity of ccRCC to sunitinib treatment, and the combination of DMF and sunitinib dramatically inhibited the cell proliferation and tumor growth of ccRCC, providing a potential therapeutic strategy to overcome sunitinib resistance in ccRCC.

Several studies have highlighted the critical role of post-translational modifications (PTMs) in regulating protein stability. For instance, D-mannose induces phosphorylation of PD-L1, which disrupts its glycosylation and triggers ubiquitin-dependent degradation [40, 41]. Similarly, acetylation of FGL1 enhances its ubiquitination and degradation [40, 41]. In this study, we elucidated the molecular mechanism by which DMF, a metabolite, can promote succination and subsequent ubiquitin-proteasome-mediated degradation of HNF1B via OTUD3, leading to destabilization and inactivation of YAP. These findings provide new evidence for metabolites regulating protein stability and cell signaling via PTMs.

In summary, we report a previously unrecognized PTM and function of HNF1B in ccRCC, and provide a potential drug and strategy for clinical treatment of ccRCC by targeting HNF1B-YAP axis.

## Materials and methods

### Cell culture and cell transfection

The cell lines 786-O, RCC4, SW620, Hela, T24, A375 and HEK-293T cells were maintained in DMEM (Meilun Biotechnology, China). A549 cells were cultured in F-12 medium, while H1299 and KTC-1 cells were maintained in RPMI 1640. All media were supplemented with 10% fetal bovine serum (FBS) (Biological Industries, Israel) and 1% penicillin/streptomycin (P/S). For cell transfection, EZ Trans (Life-iLab, China) was used according to manufacturer’s protocols.

### Reagents and antibodies

DMF (624-49-7), CCK-8 (C0005), sunitinib (557795-19-4), MLN4924 (905579-51-3) were purchased from TargetMol (Shanghai, China). MG132 (HY-13259) was obtained from MCE (New Jersey, USA). Antibodies were listed as follows: HNF1B (12533-1-AP, Proteintech), Actin (81115-1-RR, Proteintech,), GAPDH (60004-1-Ig, Proteintech), Flag-tag (HOA012FL01, AbHO), HA-tag (HOA012HA01, AbHO), YAP (ET1608-30, HUABIO), 2SC (crb2005017, Discovery Antibodies), pYAP (ET1611-69, HUABIO), mouse secondary antibody (L3032, SAB), rabbit secondary antibody (L3012, SAB).

### Mutagenesis and gene knockout

Human cDNAs of protein HNF1B were cloned into pLVX-2Flag lentiviral expression vector. Various site-directed mutants of HNF1B were generated by PCR using KOD Fx (TOYOBO, Japan). HNF1B plasmids were amplified, and the products were digested with DpnI enzyme (Takara, Japan) before being transformed into NcmDH5-α (NCM Biotech, China) for amplification. HNF1B knockout cells were generated through the CRISPR/Cas9 system. HEK-293T cells were co-transfected with LentiCRISPRv2, psPAX2, and pMD2.G in a ratio of 4:3:1. The supernatant containing virus particles was collected twice, at 48 and 72 h post transfection, and then filtered using 0.45 μm filter. For lentiviral infection, cells were incubated in lentivirus-containing medium with polybrene (10 μg/mL) for 48 h and then selected by puromycin for 7 days. The guide RNA (gRNA) sequence targeting HNF1B were as follows: 5′- AGGGCTGCTAAAATGATCAA -3; 5′- GACGTACCAGGTGTACAGAG -3.

### qPCR analysis

Total RNA was extracted using EZ-press RNA purification Kit (EZ Bioscience, USA). 1 μg of purified RNA was reverse transcribed into cDNA using 4× Reverse Transcription Master Mix (EZ Bioscience). qPCR was performed with 2× SYBR qPCR Mix (KTSM, AlpaLife) using an Applied Biosystems 7300 Plus Sequence Detection System. The human ACTIN gene was utilized for normalization. Primers used in qPCR analysis are listed below: ACTIN (5′-GGCATAGAGGTCTTTACGGATGTC-3′; 5′-TATTGGCAACGAGCGGTTCC-3′); HNF1B (5′-GGCAATTGCACAAATGTCCTCT-3′; 5′-ATTGTCTGAGGTGCCAGCAG-3′); CYR61(5′-AAGAAACCCGGATTTGTGAG-3′; 5′- GCTGCATTTCTTGCCCTTT-3′); AXL (5′-GTGGGCAACCCAGGGAATATC-3′; 5′-GTACTGTCCCGTGTCGGAAAG-3′); MYC (5′-GGCTCCTGGCAAAAGGTCA-3′; 5′-CTGCGTAGTTGTGCTGATGT-3′).

### Western blot and coimmunoprecipitation

Cells were washed with ice-cold PBS and lysed with 0.5% NP-40 lysis buffer containing 1% protease inhibitor and 1% phosphatase inhibitor at 4 °C for 30 min. Cell lysates were heated with Sodium Dodecyl Sulfate Polyacrylamide Gel Electrophoresis (SDS-PAGE) sample loading buffer at 100 °C for 10 min and then subjected to Western blot according to standard protocol. Briefly, protein samples were separated by SDS-PAGE, transferred onto nitrocellulose filter membranes and incubated with primary antibodies overnight at 4 °C, followed by HRP-conjugated anti-mouse secondary antibodies or anti–rabbit secondary antibodies at room temperature for one hour. Images were captured using Tanon 5200 imaging system (Tanon, China). For co-IP, cell lysates were collected and centrifuged at 12,000 × *g* for 15 min at 4 °C. The supernatants were incubated with anti-Flag (AbHO) beads overnight at 4 °C. The following day, beads were washed five times with NP-40 buffer, heated with SDS-PAGE sample loading buffer, and then subjected to western blot analysis as description above.

### Cell proliferation and colony formation assays

Cell proliferation was assessed via CCK-8 assay. Cells were seeded in 96-well plates at a density of 1000 cells/well, and the cell viability was measured four times with 24-h intervals by CCK-8 using a microplate reader at optical density (OD) of 450 nm. For colony formation assay, a total of 1000 cells were seeded into per well of 6-well plate and cultured for 14 days, with media changed every other day. Subsequently, colonies were washed using PBS, fixed in 10% methanol at room temperature for 15 min, and stained with 0.1% crystal violet for 30 min. After washing with PBS, images of the colonies were captured. Decolorization was performed using 33% acetic acid, and the resulting solution was analyzed to measure OD value at 570 nm for statistical analysis.

### Animal experiments

All animal experiments were approved by the Animal Care and Use Committee at Fudan University. Five-week-old male NSG mice were purchased from Model Organisms Center, Shanghai, China. The mice were housed in specific pathogen-free conditions at temperature of 22–23 °C, with free food and water access in a 12 h light/dark cycle. A total of 1 × 10^7^ 786-O cells in 50% matrigel were injected subcutaneously into mice. Once the tumors became palpable, the mice were randomly assigned into four groups and treated with different drugs by oral gavage: (1) control group (0.1% DMSO); (2) DMF group (30 mg/kg of DMF everyday); (3) sunitinib group (20 mg/kg of sunitinib every other day); (4) combined treatment group (30 mg/kg of DMF every day and 20 mg/kg of sunitinib every other day). Tumor growth was measured every four days by calipers, and tumor volume was estimated using the formula: 0.5 × L (longer diameter) × W^2^ (shorter diameter). At the experimental endpoint of experiments, the mice were euthanized, and the tumors were excised and weighed.

## Supplementary information

uncropped western blots

## Data Availability

Further information and requests for reagents may be directed to, and will be fulfilled by, the author Yanping Xu (yanpingxu@tongji.edu.cn).
