# Correction: Dimethyl fumarate promotes the degradation of HNF1B and suppresses the progression of clear cell renal cell carcinoma

**DOI:** 10.1038/s41419-025-07675-0

**Published:** 2025-05-19

**Authors:** Yue Dai, Hongchen Li, Shiyin Fan, Kai Wang, Ziyi Cui, Xinyu Zhao, Xue Sun, Mingen Lin, Jiaxi Li, Yi Gao, Ziyin Tian, Hui Yang, Bingbing Zha, Lei Lv, Yanping Xu

**Affiliations:** 1 Fifth People’s Hospital of Shanghai, MOE Key Laboratory of Metabolism and Molecular Medicine, Department of Biochemistry and Molecular Biology, School of Basic Medical Sciences, Fudan University, Shanghai, China; 2 Tongji Hospital, Frontier Science Center for Stem Cell Research, School of Life Sciences and Technology, Tongji University, Shanghai, China; 3 Department of Endocrinology, Fifth People’s Hospital of Shanghai, Fudan University, Shanghai, China; 4 Department of Neurosurgery, Huashan Hospital, Institute for Translational Brain Research, MOE Frontiers Center for Brain Science, Shanghai Medical College, Fudan University, Shanghai, China

**Keywords:** Urological cancer, Drug discovery, Ubiquitylation, Cancer metabolism

Correction to: *Cell Death and Disease* 10.1038/s41419-025-07412-7, published online 6 February 2025

In the original publication, a wrong verion of Figure 1F (sgcontrol) was incorrectly inserted. This correction does not affect the conclusions of the article.

**Amended fig1f** 
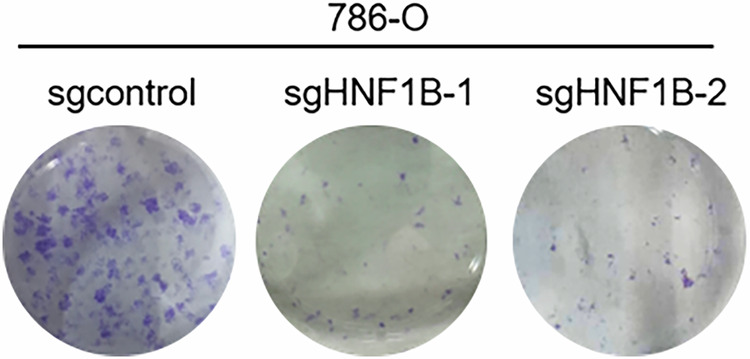


**Amended sgcontrol** 
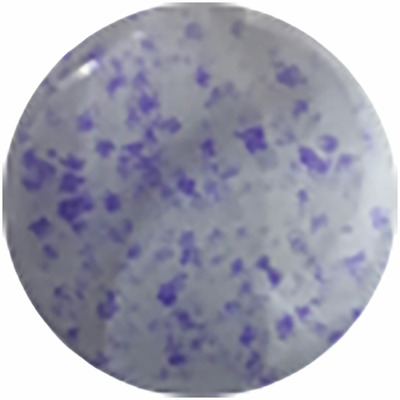


**Original fig1f** 
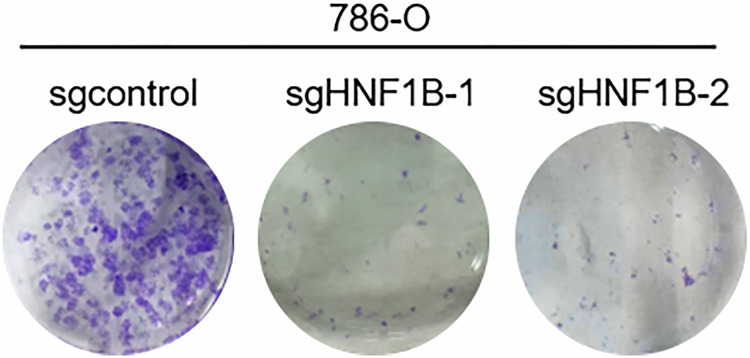


**Original sgcontrol** 
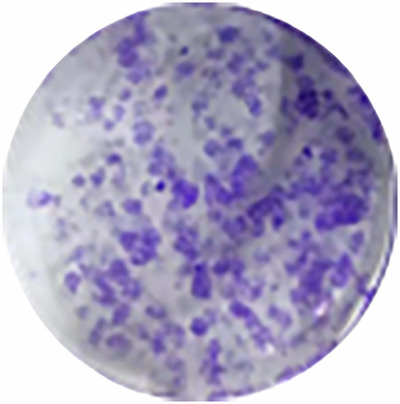


The original article has been corrected.

