# Supplementary material for: Dimethyl fumarate promotes the degradation of HNF1B and suppresses the progression of clear cell renal cell carcinoma
Source: Cell Death Dis. 2025 Feb 6;16(1):71. doi: 10.1038/s41419-025-07412-7 (PMC11802756; doi:10.1038/s41419-025-07412-7)

Fgure 1C

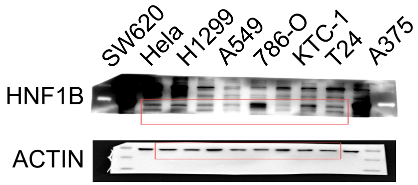

Fgure 1D

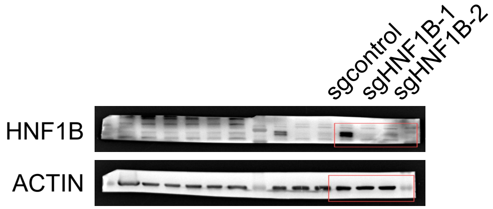

Fgure 2C

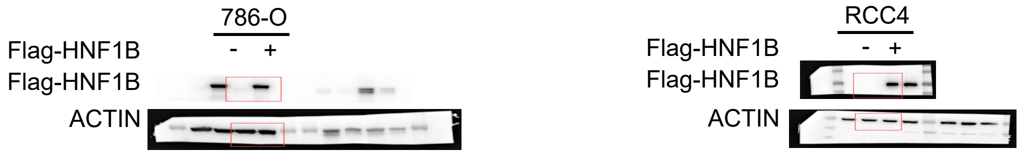

Fgure 2D

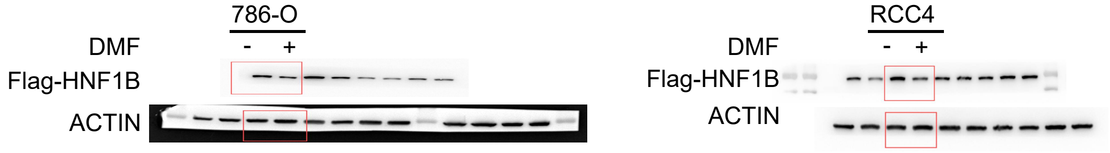

Fgure 2E

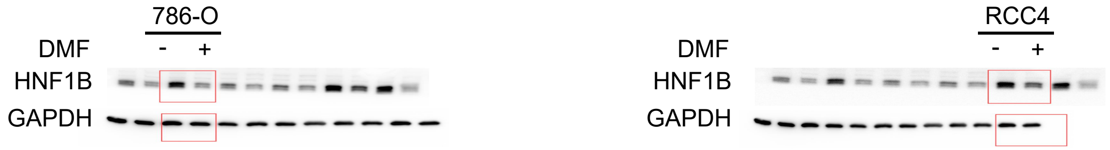

Fgure 2F

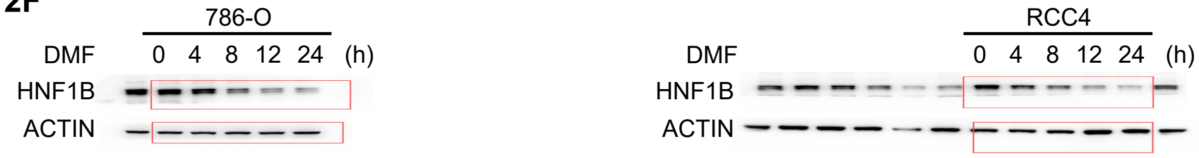

Fgure 2G

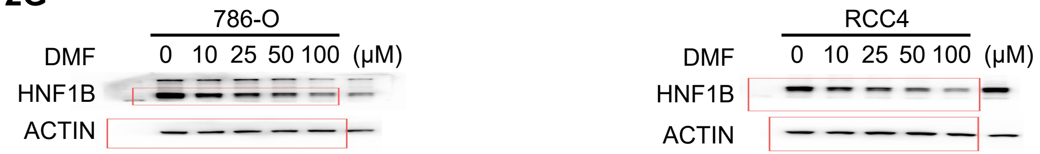

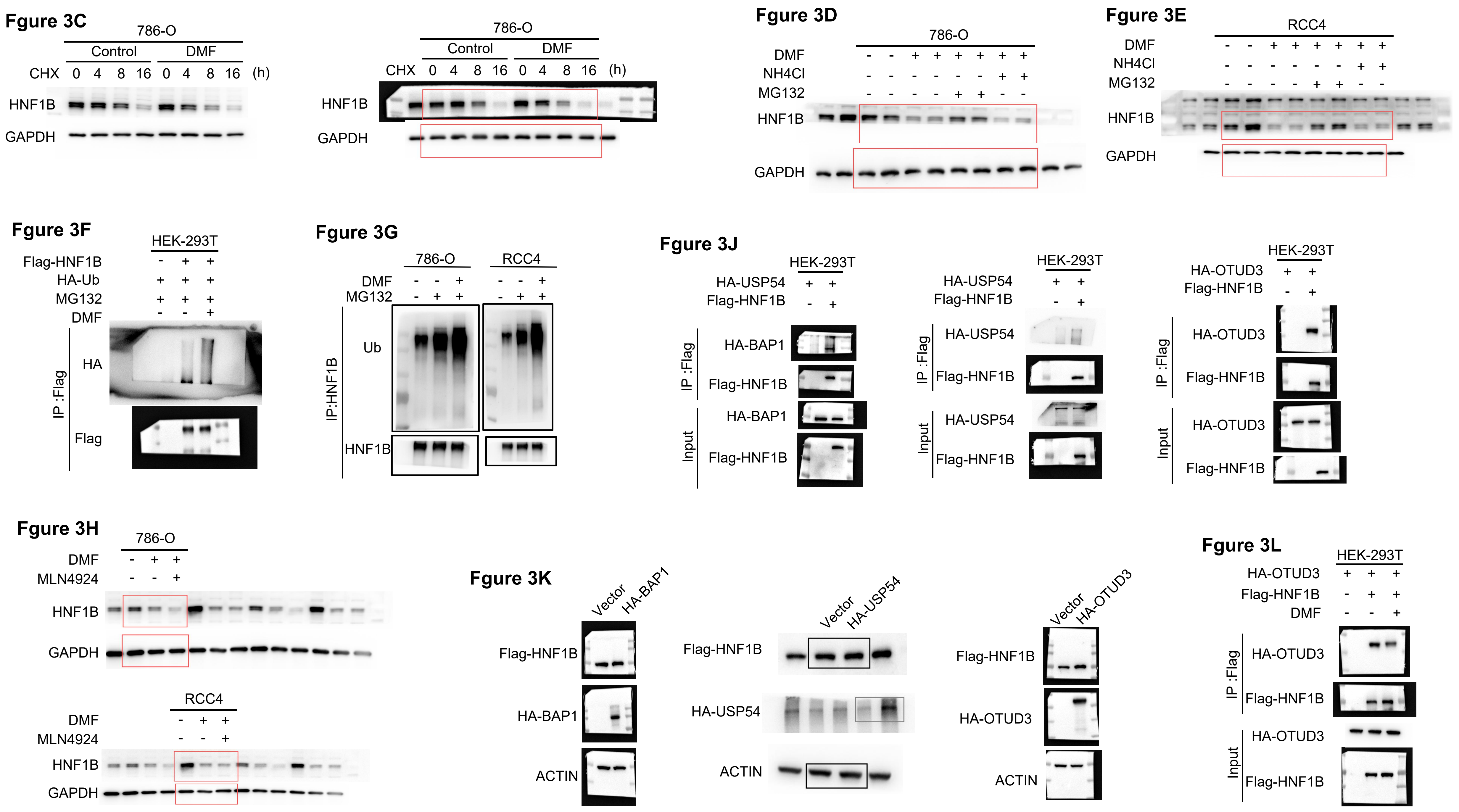

Fgure 3C
786-O
Control
DMF
CHX 0 4 8 16 0 4 8 16 (h)
HNF1B
GAPDH
Fgure 3D
786-O
DMF
NH4Cl
MG132
HNF1B
GAPDH
Fgure 3E
RCC4
DMF
NH4Cl
MG132
HNF1B
GAPDH
Fgure 3F
HEK-293T
Flag-HNF1B
HA-Ub
MG132
DMF
IP :Flag
HA
Flag
Fgure 3G
786-O
RCC4
DMF
MG132
IP:HNF1B
Ub
HNF1B
Fgure 3J
HEK-293T
HA-USP54
Flag-HNF1B
IP :Flag
HA-BAP1
Flag-HNF1B
Input
HA-BAP1
Flag-HNF1B
HA-USP54
HA-OTUD3
Fgure 3H
786-O
DMF
MLN4924
HNF1B
GAPDH
RCC4
Fgure 3K
Vector
HA-BAP1
HA-USP54
HA-OTUD3
Flag-HNF1B
ACTIN
Fgure 3L
HEK-293T
HA-OTUD3
Flag-HNF1B
DMF
IP :Flag
Input

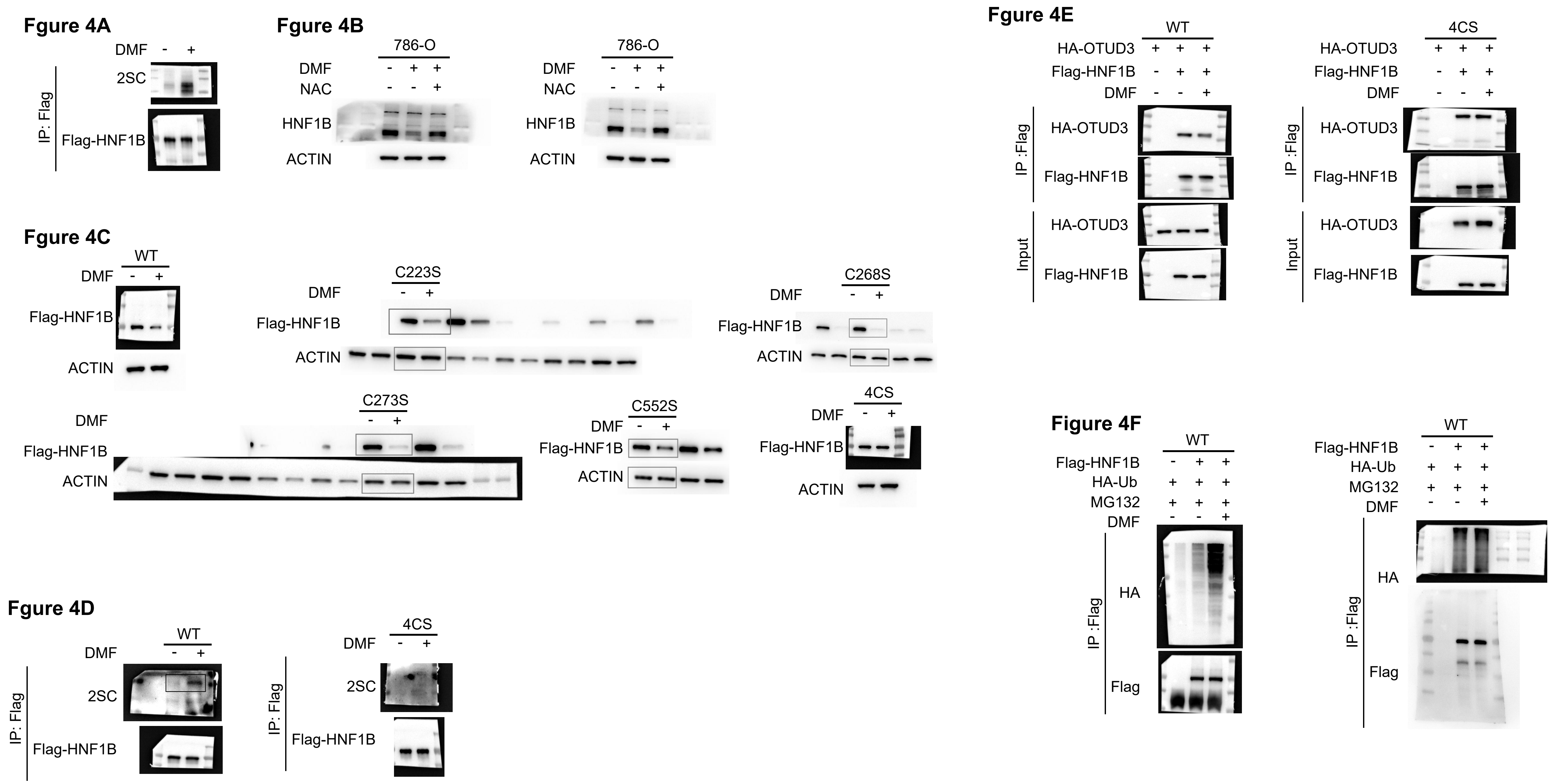

Fgure 4A
DMF - +
2SC
IP: Flag
Flag-HNF1B
Fgure 4B
786-O
DMF - + +
NAC - - +
HNF1B
ACTIN
786-O
DMF - + +
NAC - - +
HNF1B
ACTIN
Fgure 4C
WT
DMF - +
Flag-HNF1B
ACTIN
C223S
DMF - +
Flag-HNF1B
ACTIN
C268S
DMF - +
Flag-HNF1B
ACTIN
C273S
DMF - +
Flag-HNF1B
ACTIN
C552S
DMF - +
Flag-HNF1B
ACTIN
4CS
DMF - +
Flag-HNF1B
ACTIN
Fgure 4D
WT
DMF - +
2SC
IP: Flag
Flag-HNF1B
4CS
DMF - +
2SC
IP: Flag
Flag-HNF1B
Fgure 4E
WT
HA-OTUD3 + + +
Flag-HNF1B - + +
DMF - - +
IP :Flag
HA-OTUD3
Flag-HNF1B
Input
HA-OTUD3
Flag-HNF1B
4CS
HA-OTUD3 + + +
Flag-HNF1B - + +
DMF - - +
IP :Flag
HA-OTUD3
Flag-HNF1B
Input
HA-OTUD3
Flag-HNF1B
Figure 4F
WT
Flag-HNF1B - + +
HA-Ub + + +
MG132 + + +
DMF - - +
IP :Flag
HA
Flag
WT
Flag-HNF1B - + +
HA-Ub + + +
MG132 + + +
DMF - - +
IP :Flag
HA
Flag

Figure 5A

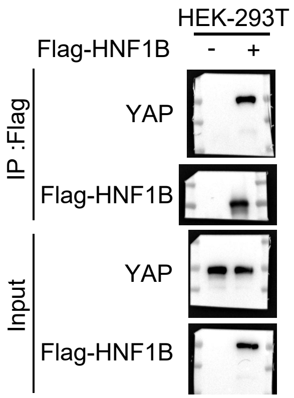

Figure 5B

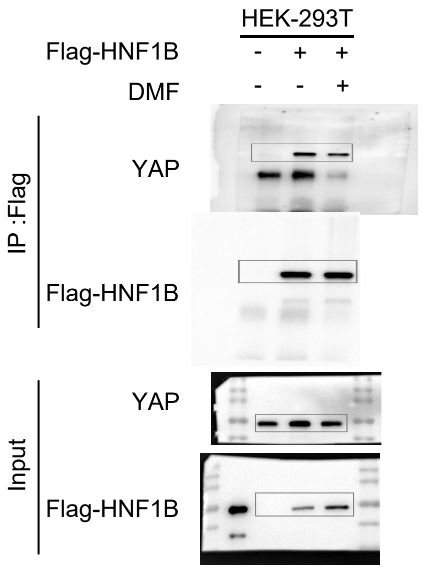

Figure 5C

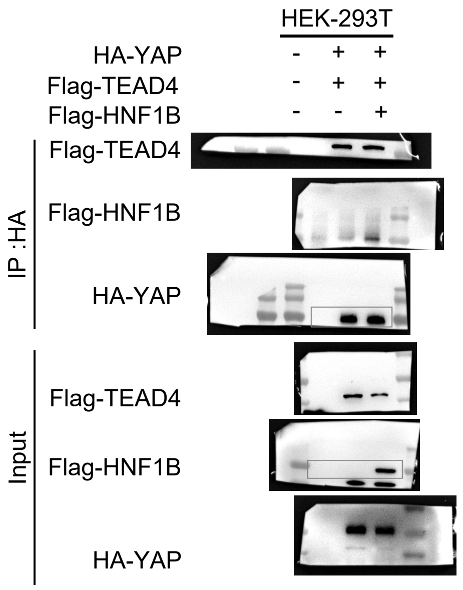

Figure 5D

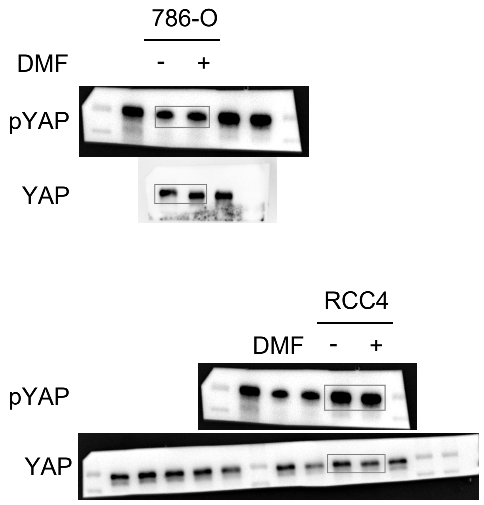

Figure 5E

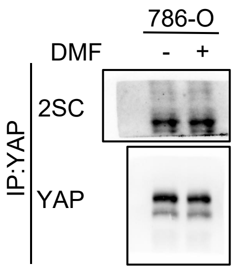

Figure 5F

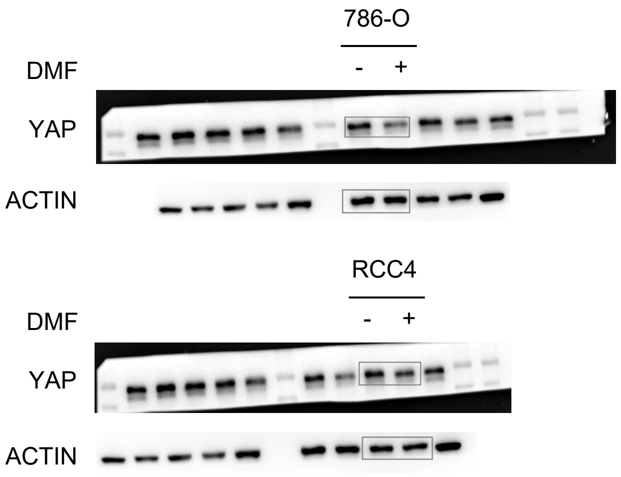

Figure 5K

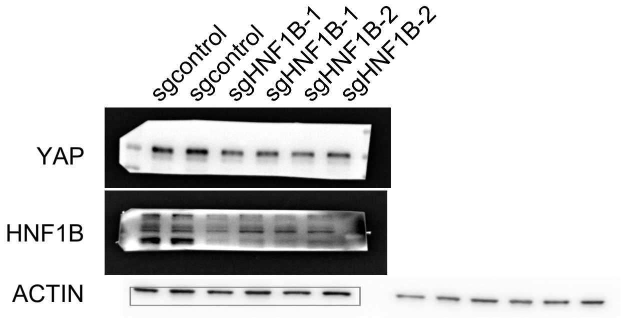

Figure 5P

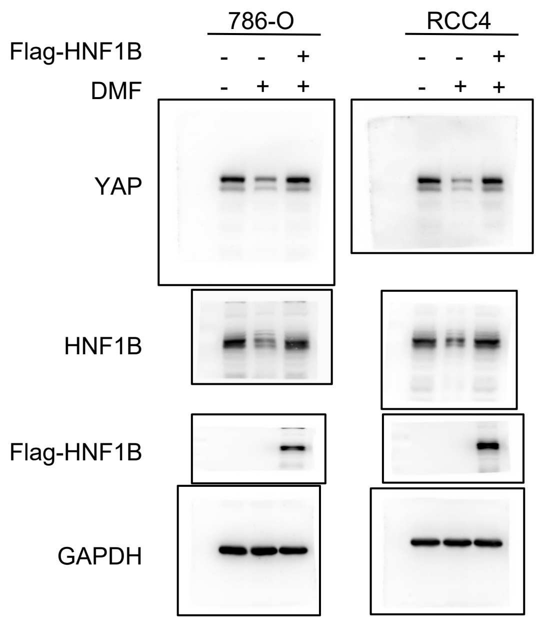

Figure 5Q

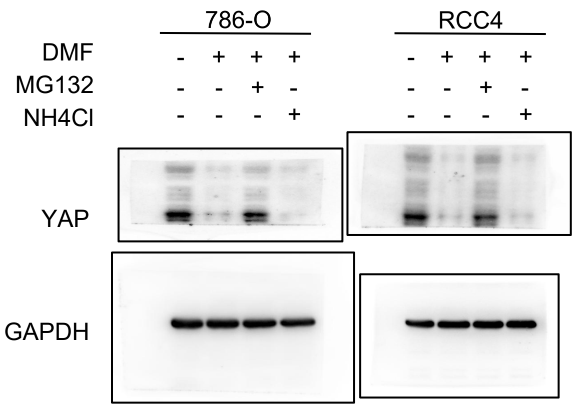

Figure 5R

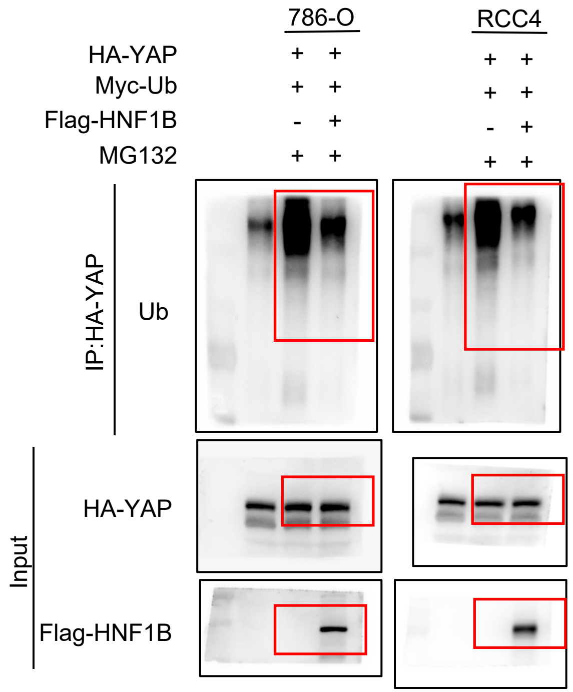

Figure 5S

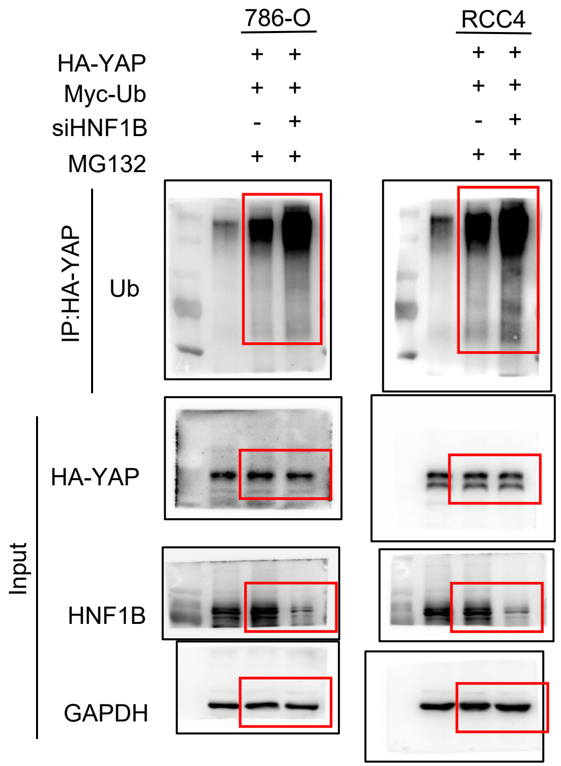

Supplement: Supplementary file 1 — uncropped western blots [file 41419_2025_7412_MOESM1_ESM.pdf]
